# Prognostic Nomograms Based on Ground Glass Opacity and Subtype of Lung Adenocarcinoma for Patients with Pathological Stage IA Lung Adenocarcinoma

**DOI:** 10.3389/fcell.2021.769881

**Published:** 2021-12-08

**Authors:** Wenyu Zhai, Dachuan Liang, Fangfang Duan, Wingshing Wong, Qihang Yan, Li Gong, Renchun Lai, Shuqin Dai, Hao Long, Junye Wang

**Affiliations:** ^1^ Department of Thoracic Surgery, State Key Laboratory of Oncology in South China, Collaborative Innovation Center for Cancer Medicine, Sun Yat-sen University Cancer Center, Guangzhou, China; ^2^ Department of Medical Oncology, State Key Laboratory of Oncology in South China, Collaborative Innovation Center for Cancer Medicine, Sun Yat-sen University Cancer Center, Guangzhou, China; ^3^ Department of Thoracic Surgery, The Second Affiliated Hospital Zhejiang University School of Medicine, Hangzhou, China; ^4^ Department of Anaesthesiology, State Key Laboratory of Oncology in South China, Collaborative Innovation Center for Cancer Medicine, Sun Yat-sen University Cancer Center, Guangzhou, China; ^5^ Department of Laboratory Medicine, State Key Laboratory of Oncology in South China, Collaborative Innovation Center for Cancer Medicine, Sun Yat-sen University Cancer Center, Guangzhou, China

**Keywords:** lung adenocarcinoma, pathological subtype, GGO (ground glass opacity), nomogram, prognosis

## Abstract

The value of lung adenocarcinoma (LUAD) subtypes and ground glass opacity (GGO) in pathological stage IA invasive adenocarcinoma (IAC) has been poorly understood, and reports of their association with each other have been limited. In the current study, we retrospectively reviewed 484 patients with pathological stage IA invasive adenocarcinoma (IAC) at Sun Yat-sen University Cancer Center from March 2011 to August 2018. Patients with at least 5% solid or micropapillary presence were categorized as high-risk subtypes. Independent indicators for disease-free survival (DFS) and overall survival (OS) were identified by multivariate Cox regression analysis. Based on these indicators, we developed prognostic nomograms of OS and DFS. The predictive performance of the two nomograms were assessed by calibration plots. A total of 412 patients were recognized as having the low-risk subtype, and 359 patients had a GGO. Patients with the low-risk subtype had a high rate of GGO nodules (*p* < 0.001). Multivariate Cox regression analysis showed that the high-risk subtype and GGO components were independent prognostic factors for OS (LUAD subtype: *p* = 0.002; HR 3.624; 95% CI 1.263–10.397; GGO component: *p* = 0.001; HR 3.186; 95% CI 1.155–8.792) and DFS (LUAD subtype: *p* = 0.001; HR 2.284; 95% CI 1.448–5.509; GGO component: *p* = 0.003; HR 1.877; 95% CI 1.013–3.476). The C-indices of the nomogram based on the LUAD subtype and GGO components to predict OS and DFS were 0.866 (95% CI 0.841–0.891) and 0.667 (95% CI 0.586**–**0.748), respectively. Therefore, the high-risk subtype and GGO components were potential prognostic biomarkers for patients with stage IA IAC, and prognostic models based on these indicators showed good predictive performance and satisfactory agreement between observational and predicted survival.

## Introduction

Lung cancer is the leading cause of tumor-related death worldwide and lung adenocarcinoma (LUAD) has been the main histological type (Lortet-Tieulent et al., 2014; Sung et al., 2021). For stage IA non-small cell lung cancer, patients have satisfactory long-term survival after radical surgery, and the 5-year overall survival (OS) rate is 80–90% (Goldstraw et al., 2016). Especially for patients with adenocarcinoma *in situ* (AIS) and minimally invasive adenocarcinoma (MIA), their 5-year OS rate is nearly 100% (Cohen et al., 2018; Yotsukura et al., 2021). However, there are still approximately 20% of patients who die from recurrence. It is not difficult to conclude that basically all recurrences are concentrated in cases of invasive NSCLC, showing that stage IA NSCLC is a group with heterogeneity, and it is necessary to identify patients with high risk of recurrence with invasive adenocarcinoma (IAC) who might require closer follow-up and even adjuvant therapy.

The International Association for the Study of Lung Cancer/American Thoracic Society/European Respiratory Society introduced a novel international multidisciplinary classification of lung adenocarcinoma in 2011 (Travis et al., 2011), which classified IAC using 5 five major histopathological patterns including lepidic, acinar, papillary, solid, micropapillary, and 4 variants. This classification was also adopted by the World Health Organization in 2015 (Travis et al., 2015). Patients with solid or micropapillary predominant subtypes have been proven to suffer from a worse clinical outcome in several studies (Tsuta et al., 2013; Yanagawa et al., 2014; Ujiie et al., 2015). In fact, mixtures of the histologic pattern are common in IACs. The presence of solid and/or micropapillary features with no predominant pattern can be associated with poor prognosis. Yanagawa et al. reported that patients with minor solid or micropapillary patterns present suffered from a high risk of recurrence (Yanagawa et al., 2016).

Ground glass opacity (GGO) is defined as a hazy opacity not obscuring the underlying pulmonary vessels or bronchial structures in the lung window (Austin et al., 1996). It is widely accepted that the GGO component is a positive prognostics factor for patients with LUAD (Hattori et al., 2017a; Berry et al., 2018). Hattori et al. reviewed 497 patients with clinical stage IA IAC and found even a small proportion of GGO components was related to prolonged OS (Hattori et al., 2017b).

Although prognostic nomograms including sex, age, operative approach, examined lymph nodes, vascular invasion, and EGFR gene mutation for stage IA NSCLC have been developed (Merritt et al., 2020; Yang et al., 2020; Cai et al., 2021), a prognostic model based on LUAD subtype and GGO component especially designed for stage IA IAC has been lacking. Recently, researchers have focused on the relationship between GGO component and LUAD subtypes as well as the differential gene mutation profiles among different LUAD subtypes and CT characteristics (Gao et al., 2017). This information is useful to guiding treatment decisions, but related reports remain inadequate. Therefore, we aimed to identify the impact of LUAD subtypes and GGO components in patients with stage IA IAC and develop a nomogram based on them. We also explored the relationship between GGO components and LUAD subtypes and the differential gene mutation profiles among patients.

### Patients and Methods

#### Patients

Patients with pathological IA LUAD who accepted radical resection between January 2012 and August 2018 at the Sun Yat-sen University Cancer Center (SYSUCC) were retrospectively reviewed in this study. This study was approved by the Institutional Review Board of SYSUCC (IRB No. SZR2019-108) and we conducted the current study following the Declaration of Helsinki. The written informed consent for this retrospective study was waived due to the retrospective nature of our study.

In this study, the tumor pathologic staging was based on the AJCC staging, 8th edition (Ettinger et al., 2019). The key inclusion criteria were as follows: (1) pathological diagnosis of stage IA LUAD; (2) confirmed negative surgical margin (R0); (3) pathological evaluation based on the IASLC/ATS/ERS lung adenocarcinoma classification system. Patients who met the following criteria were excluded: (1) received neoadjuvant therapy; (2) multiple primary tumors; (3) death within 1 month after surgical resection; and (4) pathological diagnosis as adenocarcinoma in situ (AIS) and minimally invasive adenocarcinoma (MIA).

### Pathological Evaluation and Defining of High-Risk Subtype

All surgical specimens were processed by formalin fixation immediately after surgery. Then, the dehydrated specimens were processed by paraffin embedding. The specimens were processed by hematoxylin–eosin (HE) staining after the paraffin slices were dewaxed. Further pathological evaluation was performed by two pathologists who were blinded to the clinical information. According to the new WHO classification, pathological assessment using the semiquantitative estimation of all patterns of 5% increment and each tumor was categorized into the following subtypes: lepidic, acinar, papillary, solid, micropapillary, and variants of invasive adenocarcinoma (Travis et al., 2015). The pattern with the greatest percentage was defined as predominant pattern. Two or more patterns with the same percentage were defined as a mixed pattern. A non-predominant pattern was the subtype with no less than 5% but not reaching predominance. The high-risk subtype was defined as any subtype with at least 5% solid or micropapillary presence.

### Radiological Evaluation and Gene Testing Methods

Thin slice CT was used to measure the GGO and consolidation component and the images were reviewed by two radiologists independently. Tumor size was defined as the maximum diameter on the axial plane in the lung window, and solid tumor size was defined as maximum diameter of the solid component. Consolidation-to-tumor ratio (CTR) was defined as the ratio of the solid tumor size to the tumor size. DNA extraction from paraffin-embedded tumor tissue was performed using a QIAGEN DNA FFPE Kit (Qiagen, Dusseldorf, Germany) according to the instructions of the manufacturer and quantification was conducted using a NanoDrop 2000 (NanoDrop Technologies, Wilmington, DE). The mutations of EGFR gene from exons 18, 19, 20, and 21 was detected by an EGFR Mutations Detection Kit (SINOMD, Beijing, China). Amplification refractory mutation system-polymerase chain reaction (ARMS-PCR) was conducted using ABI 7500 (Applied Biosystems, Foster City, CA). After dewaxing and dehydration of the paraffin embedded sections, EML4-ALK fusion was screened with fluorescent *in situ* hybridization (FISH) performing on 100 nuclei by Vysis ALK Break Apart FISH Probe Kit (Abbott Molecular, Des Plaines, IL). A sample was considered positive for an ALK rearrangement when 15% or greater of nuclei showed a split orange and green signal and/or an isolated (single) orange signal. The results of gene testing were obtained from reports of molecular diagnosis.

### Follow-Up and Endpoints

Regular follow-up was performed at 3-month intervals for the first 2 years, every 6 months until 5 years, and per year in subsequent years, mainly including blood tests for detection of tumor markers of lung cancer and chest and abdominal computed tomography (CT) scans. Brain magnetic resonance imaging (MRI), bone scintigraphy, and positron emission tomography were performed if necessary.

The main endpoints of this study were the overall survival time (OS) and the disease-free survival time (DFS). The DFS was defined as the date of the surgery to the date of the first event recurrence or death, and the OS was calculated from the date of operation to the date of death or the last follow-up.

### Statistical Analysis

Continuous data are shown as the mean ± SD or median and were compared using Student’s *t*-test. Categorical variables were tested using the chi-square (χ^2^) or the Mann-Whitney U test. Survival curves were calculated by the Kaplan-Meier method and compared with the log-rank test. Variables with a *p* value less than 0.1 in the univariate analysis were further entered into the multivariate Cox analysis, independent factors from which were integrated to develop prognostic models using R packages “rms”. The predictive performance of the prognostic nomogram was assessed by calculating Harrell’s concordance index (C-index). In addition, we performed calibration curves to evaluate its discriminative accuracy at 3 and 5 years. All statistical analyses were performed using SPSS software version 22.0 for Windows (SPSS Inc., Chicago, IL) and R software (version 4.0.3; http://www.r-project.org). Statistical significance was defined as *p* < 0.05 and the reported significance levels were all two-sided.

## Results

### Patient Characteristics

**FIGURE 1:**
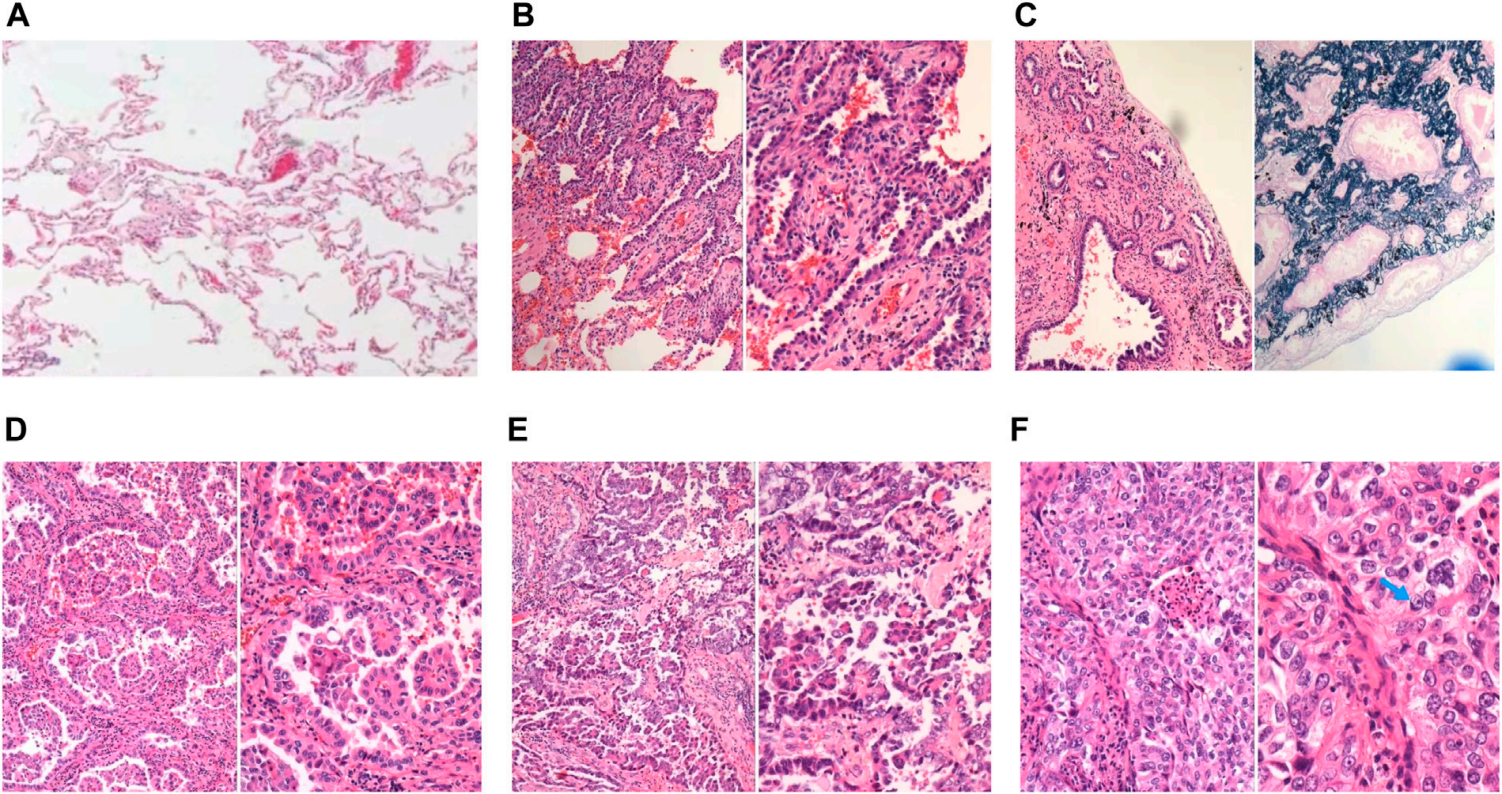
Representative images of normal lung tissue and 5 major histopathological patterns. **(A)** Normal lung tissue in 40x light microscope; **(B)** left: lepidic patterns in 100x microscope, right: lepidic patterns in 200x microscope; **(C)** left: acinar patterns in 100x microscope, right: acinar patterns with elastic-fiber staining in 200x microscope; **(D)** left: papillary patterns in 100x microscope, right: papillary patterns in 200x microscope; **(E)** left: micropapillary patterns in 100x microscope, right: micropapillary patterns in 200x microscope; **(F)** left: solid patterns in 200x microscope, right: solid patterns in 400x microscope, blue arrow: solid pattern cancer cell with mucin.

**FIGURE 2:**
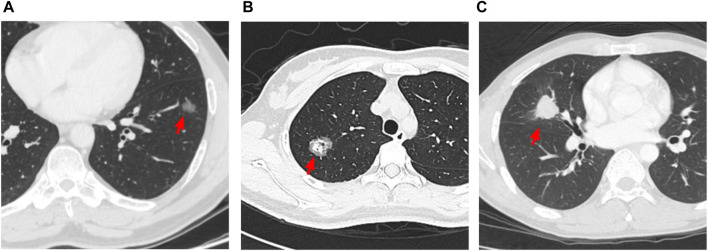
Representative images of 3 different characteristics of CT. **(A)** red arrow: pure GGO nodule with diameter of 1.6 cm; **(B)** red arrow: part GGO nodule with diameter of 2.6 cm; **(C)** red arrow: pure solid nodule with diameter of 2.6 cm.

A total of 484 patients were included in this study. The baseline characteristics are shown in Table 1 **.** The pathological subtype of the patients included lepidic predominant (*n* = 121; 25%), acinar predominant (*n* = 244; 50.4%), papillary predominant (*n* = 53; 11%), solid predominant (*n* = 18; 3.17%), micropapillary predominant (*n* = 4; 0.8%), variants (*n* = 14; 2.9%), and mixed subtype (*n* = 30; 6.2%). Representative images of 5 major histopathological patterns are shown in Figure 1. The CT characteristics of the patients included pure GGO (*n* = 92; 19%), part GGO (*n* = 267; 55.2%), and pure solid (*n* = 125; 25.8%). Representative images of pure GGO, part GGO, and pure solid are shown in Figure 2. A total of 412 patients were defined as low-risk subtype and 72 patients were defined as high-risk subtype, while 359 patients had a nodule with GGO component, and 125 patients had a pure solid nodule.

**TABLE 1:** Patient’s characteristics.

Characteristics	Media or case NO. (%)
Gender	
Male	248 (51.2)
Female	236 (48.8)
Age (year)	61 ± 9.7
<60	229 (47.3)
60–70	202 (41.7)
>70	53 (11)
Tumor size (cm)	1.8 ± 0.6
Smoking history	
No	320 (66.1)
Yes or ever	164 (33.9)
8th TNM stage	
IA1	51 (10.5)
IA2	251 (51.9)
IA3	182 (37.6)
Tumor location	
Right upper lobe	162 (33.5)
Right middle lobe	44 (9.1)
Right lower lobe	84 (17.4)
Left upper lobe	115 (23.8)
Left lower lobe	79 (16.3)
Differentiation degree	
Well	65 (13.4)
Moderate	314 (64.9)
Poor	105 (21.7)
Vascular invasion	
Positive	21 (4.3)
Negative	463 (95.7)
Operative approach	
Sublobectomy	38 (7.9)
Lobectomy	446 (92.1)
Number of N2 station examination	3 ± 1.4
Number of N1 station examination	3 ± 1.2
Thoracotomy or VATS	
Thoracotomy	128 (26.4)
VATS	356 (73.6)
EGFR gene mutation	
Negative	126 (26.0)
Positive	218 (45.0)
Unknown	140 (28.9)
ALK rearrangement	
Negative	320 (66.1)
Positive	11 (2.3)
Unknown	153 (31.6)
Pathologic subtype	
Lepidic predominant	121 (25.0)
Acinar predominant	244 (50.4)
Papillary predominant	53 (11.0)
Solid predominant	18 (3.7)
Micropapillary predominant	4 (0.8)
Variants	14 (2.9)
mixed subtype	30 (6.2)
Solid component	42 (8.7)
Micropapillary component	36 (8.7)
Solid or micropapillary component	72 (14.9)
CT characteristics	
Pure GGO	92 (19.0)
Part GGO	267 (55.2)
Pure solid	125 (25.8)

VATS, video-assisted thoracoscopic surgery; EGFR, epidermal growth factor receptor; ALK, anaplastic lymphoma kinase; CT, computed tomography; GGO, ground-glass opacity.

As shown in Table 2, patients with low-risk subtype had a higher rate of GGO components. Except from patients with unknown EGFR and ALK status, patients with low-risk subtype had a higher rate of EGFR gene mutation (68.3 vs. 37.0%, *p* < 0.001) and a lower rate of ALK rearrangement (2.2 vs. 9.6%, *p* = 0.017).

**TABLE 2:** Difference of CT characteristics and gene status between low-risk subtype and high-risk subtype.

Characteristics	Low-risk subtype *n* = 412	High-risk subtype *n* = 72	*p* Value
CT characteristics			**<0.001**
GGO components	323 (78.4)	36 (50.0)	
Pure solid	89 (21.6)	36 (50.0)	
EGFR gene mutation			**<0.001** ^a^
Negative	92 (22.3)	34 (47.2)	
Positive	198 (48.1)	20 (27.8)	
Unknown	122 (29.6)	18 (25.0)	
ALK rearrangement			**0.017** ^a^
Negative	273 (66.3)	47 (65.3)	
Positive	6 (1.5)	5 (6.9)	
Unknown	133 (32.3)	72 (27.8)	

EGFR, epidermal growth factor receptor; ALK, anaplastic lymphoma kinase; CT, computed tomography; GGO, ground-glass opacity.

a The p value was calculated excluded the patients with unknown EGFR and ALK status.

Table 3 demonstrated that patients with pure solid nodules had a higher a rate of high-risk subtype (*p* < 0.001) When excluding patients with unknown EGFR and ALK status, patients with GGO components had a higher rate of EGFR gene mutation (69.8% vs. 48.9.0%, *p* < 0.001) and a similar rate of ALK rearrangement (2.5 vs. 5.3%, *p* = 0.306). To further determine the relationship between GGO components and LUAD, we collected the CTR of the nodule in patients with high-risk subtype and found that only 3 patients had a CTR less than 0.5 (Supplementary Table S1).

**TABLE 3:** Difference of LUAD subtype and gene status between GGO and pure solid nodule.

Characteristics	GGO component *n* = 359	Pure solid *n* = 125	*p* Value
Pathologic subtype			**<0.001**
Low-risk subtype	323 (90.0)	89 (71.2)	
High-risk subtype	36 (10.0)	36 (28.8)	
EGFR gene mutation			**<0.001** ^a^
Negative	73 (20.3)	53 (42.4)	
Positive	169 (47.1)	49 (39.2)	
Unknown	117 (32.6)	18 (18.4)	
ALK rearrangement			0.306^a^
Negative	230 (64.1)	90 (72)	
Positive	6 (1.7)	5 (4.0)	
Unknown	123 (34.3)	30 (24.0)	

EGFR, epidermal growth factor receptor; ALK, anaplastic lymphoma kinase; GGO, ground-glass opacity.

a The p value was calculated excluded the patients with unknown EGFR and ALK status.

### Survival Analysis

**FIGURE 3:**
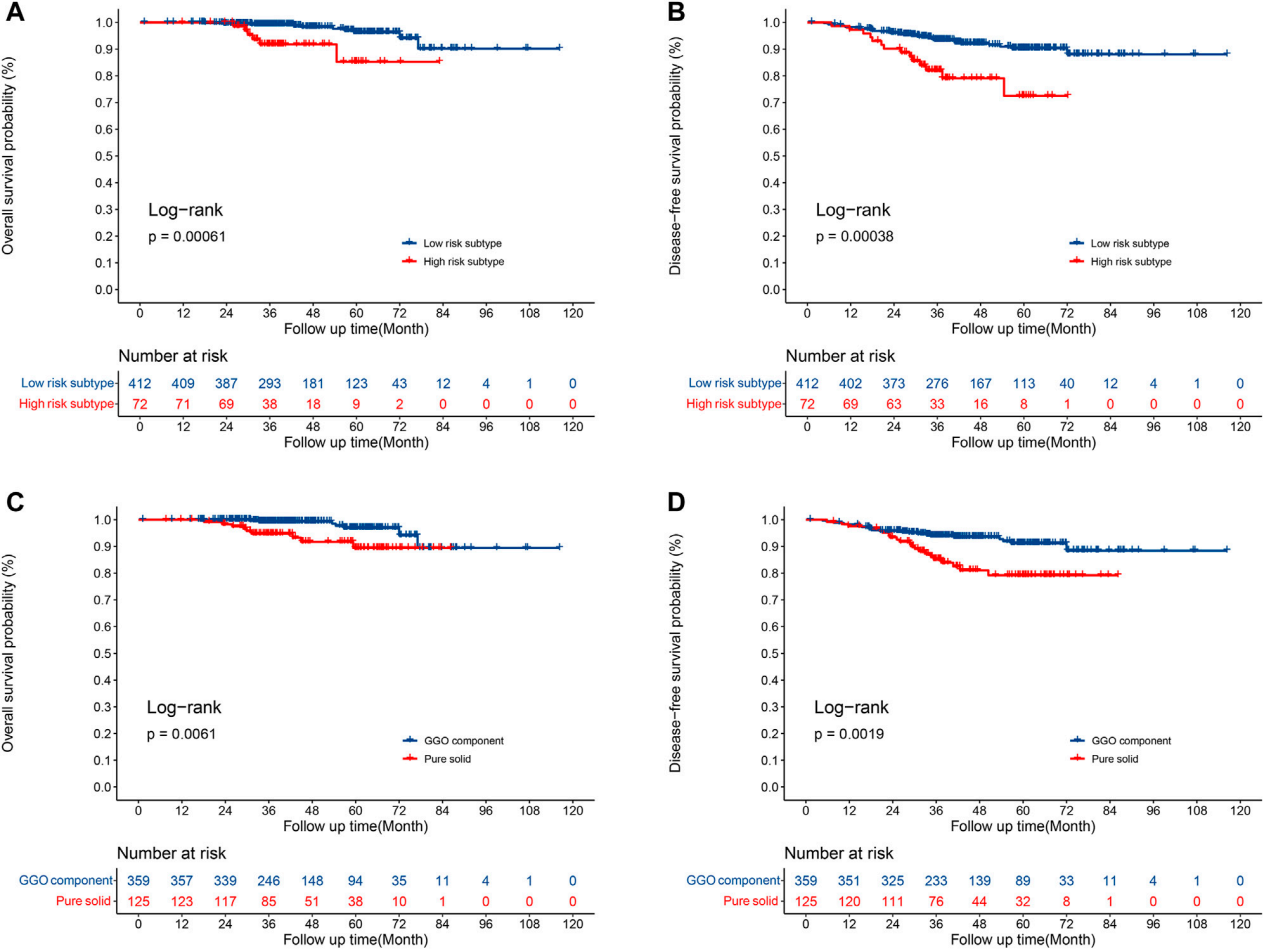
Survival for patients with low-risk and high-risk subtypes. **(A)** Overall survival; **(B)** disease-free survival. Survival for patients with GGOs and pure solid nodules. **(C)** Overall survival; **(D)** disease-free survival.

The median overall follow-up time was 42.6 months. Compared to patients with the high-risk subtype, patients with low risk had a significantly longer OS (5-year OS rate 96.3 vs. 85.2%, *p* = 0.0006; Figure 3A) and DFS (5-year DFS rate 90.3 vs. 72.4%, *p* = 0.004; Figure 3B) times. In addition, a significant improvement in the OS (5-year OS rate 96.9 vs. 89.4%, *p* = 0.0061; Figure 3C) and DFS (5-year DFS rate 91.2 vs. 79.2%, *p* = 0.0019; Figure 3D) time were observed in patients with GGO component compared to patients with pure solid nodules.

As shown in Table 4, advanced age, differentiation degree, high-risk subtype, GGO components were statistically significant in univariate analysis of OS, furthermore, multivariate Cox analysis demonstrated that advanced age (*p* = 0.001; HR 8.442; 95% CI 2.562–27.815), high-risk subtype (*p* = 0.002; HR 3.624; 95% CI 1.263–10.397), and GGO component (*p* = 0.001; HR 3.186; 95% CI 1.155–8.792) remained to be independent prognostic indicators for OS.

**TABLE 4:** Univariate and Multivariate Analysis for entire patients.

Factors	Univariate analysis	Multivariate analysis	*p* value
	*p* value	HR (95%CI)	
Analysis of OS			
Gender	0.422		
Age (year)			
<60	References		References
60–70	0.912	0.913 (0.202–4.127)	0.906
>70	**0.001**	8.442 (2.562–27.815)	**0.001**
Tumor size (cm)	0.401		
Smoking history	0.066	1.673 (0.585–4.788)	0.337
8th TNM stage			
IA1	References		
IA2	0.944		
IA3	0.946		
Differentiation degree	**0.006**	2.063 (0.837–5.087)	0.116
Vascular invasion	0.513		
High-risk subtype	**0.002**	3.624 (1.263–10.397)	**0.017**
Operative approach	0.299		
EGFR gene mutation			
Negative	References		
Positive	0.481		
Unknown	0.415		
ALK rearrangement			
Negative	References		
Positive	0.983		
Unknown	0.784		
Number of N2 stations examined	0.273		
Number of N1 stations examined	0.219		
GGO component (positive VS. negative)	**0.010**	3.186 (1.155–8.792)	**0.025**
Thoracotomy or VATS	0.241		
Analysis of DFS			
Gender	0.203		
Age (year)			
<60	References		References
60–70	0.567	0.789 (0.401–1.550)	0.491
>70	0.080	1.553 (0.717–3.363)	0.265
Tumor size (cm)	0.080	1.590 (0.969–2.611)	0.067
Smoking history	0.172		
8th TNM stage			
IA1	References		
IA2	0.446		
IA3	0.179		
Differentiation degree	**0.005**	1.455 (0.825–2.533)	0.198
Vascular invasion	0.506		
High-risk subtype	**0.001**	2.824 (1.448–5.509)	**0.002**
Operative approach	0.297		
EGFR gene mutation			
Negative	References		
Positive	0.584		
Unknown	0.179		
ALK rearrangement			
Negative	References		
Positive	0.945		
Unknown	0.554		
Number of N2 stations examined	0.892		
Number of N1 stations examined	**0.025**	0.735 (0.573–0.944)	**0.016**
GGO component (positive vs. negative)	**0.003**	1.877 (1.013–3.476)	**0.045**
Thoracotomy or VATS	0.760		

OS, overall survival; DFS, disease-free survival; VATS, video-assisted thoracoscopic surgery; EGFR, epidermal growth factor receptor; ALK, anaplastic lymphoma kinase; GGO, ground-glass opacity.

Differentiation degree, high-risk subtype, GGO components, and number of N2 stations examined were statistically significant in univariate analysis of DFS. In multivariate analysis, high-risk subtype (*p* = 0.001; HR 2.284; 95% CI 1.448–5.509), and GGO components (*p* = 0.003; HR 1.877; 95% CI 1.013–3.476) were negatively associate with DFS. The number of N1 stations examined (*p* = 0.025; HR 0.735; 95% CI 0.573–0.944) was positively associated with DFS.

### Development of the Nomogram

**FIGURE 4:**
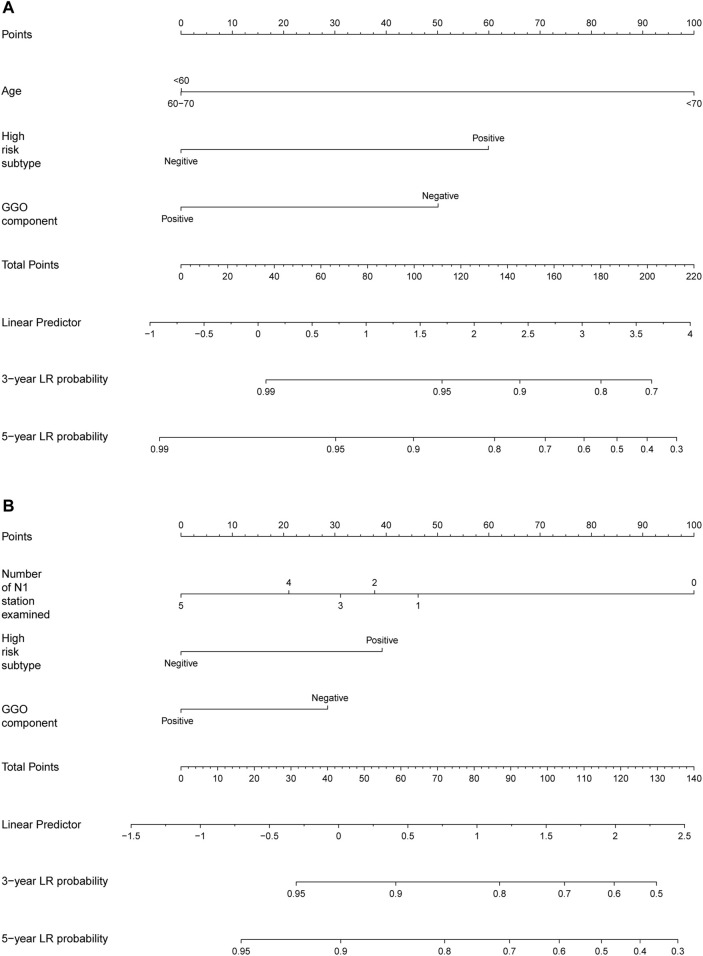
Nomogram for predicting the 3-year and 5-year survival rates in stage IA NSCLC. **(A)** Overall survival; **(B)** disease-free survival. For each patient, the scores of three factors (age, GGO component, and high-risk subtype in predicting OS; number of N1 stations examined, GGO component, and high-risk subtype in predicting DFS) are represented as points by projecting them onto the uppermost line (point scale). Totaling the three variables and projecting the total point value downward onto the bottommost line can determine the probability of 3- and 5-year OS and DFS.

Based on above identified prognostic factors from multivariate Cox regression analysis, predictive models for OS and DFS were developed and represented as graphical nomograms (Figure 4). The nomogram of OS illustrated that age, high-risk subtype, GGO components shared crucial contributions to the prognosis. The nomogram of DFS illustrated the number of N1 stations examined, high-risk subtype, GGO component sharing crucial contributions to the prognosis, which showed satisfactory predictive performance with an excellent Harrell’s C-index for DFS (0.667; 95% CI 0.586–0.748) and OS (0.866; 95% CI 0.841–0.891), respectively. The prognostic score of each factor in the nomogram and the survival probability of different total scores are provided in Supplementary Table S2.

**FIGURE 5:**
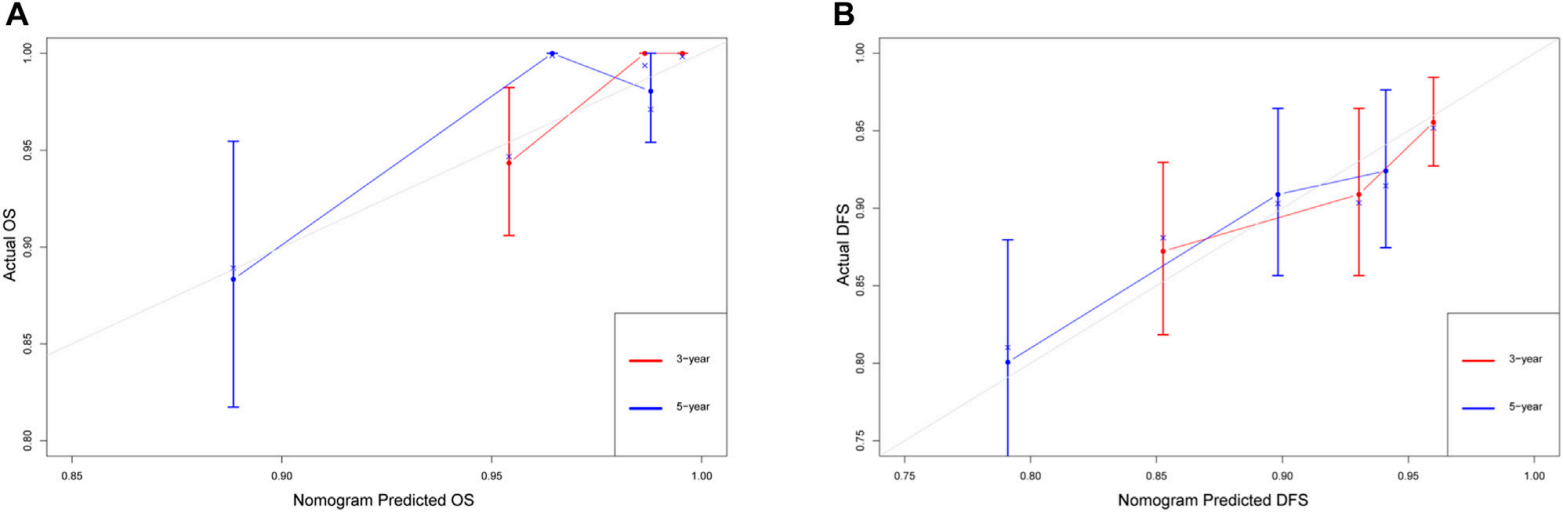
The calibration curves for predicting the 3-year and 5-year survival rates in stage IA NSCLC. **(A)** Overall survival; **(B)** disease-free survival. The x-axis represents the predicted probability of survival, the y-axis represents the actual probability of survival, and the ideal line is the diagonal of the graph. The closer that the drawn line is to the diagonal, the better that the calibration model is. *N* = 482; The error bars: the 95% CIs of actual survival.

Additionally, calibration plots of internal validation presented a good agreement between nomogram predicted and actual OS and DFS at 3, and 5 years (Figure 5). The Harrell’s C-index for the established nomogram of OS and DFS were 0.866 (95% CI 0.841–0.891) and 0.667 (95% CI 0.586–0.748), respectively.

### Interaction Analyses

As shown in Table 5, after adjusting for other factors, the interaction analysis showed an insignificant interaction effect between high-risk subtype and GGO component on the OS rate (HR _(High-risk subtype * GGO component)_ = 0.826, 95% CI 0.220–3.106, *p* = 0.778) and the DFS rate (HR _(High-risk subtype * GGO component)_ = 0.732, 95% CI 0.193–2.783, *p* = 0.647). The positive impact of high-risk subtype (HR = 2.581, 95% CI 1.332–5.002, *p* = 0.005) and GGO component (HR = 1.916, 95% CI 1.040–3.529, *p* = 0.037) on the OS rate was independent. Likewise, the positive impact of high-risk subtype (HR = 2.824, 95% CI 1.448–5.509, *p* = 0.002) and GGO component (HR = 1.877, 95% CI 1.013–3.476, *p* = 0.045) on the DFS rate was independent.

**TABLE 5:** Interaction between GGO component and high-risk subtype.

Factors	Adjusted HR^a^ (95%CI)	*p* Value
Analysis of OS		
GGO component	1.916 (1.040–3.529)	**0.037**
High-risk subtype	2.581 (1.332–5.002)	**0.005**
Interaction effect		
GGO component * High-risk subtype	0.826 (0.220–3.106)	0.778
Analysis of DFS		
GGO component	1.877 (1.013–3.476)	**0.045**
High-risk subtype	2.824 (1.448–5.509)	**0.002**
Interaction effect		
GGO component * High-risk subtype	0.732 (0.193–2.783)	0.647

OS, overall survival; DFS, disease-free survival.

a Multivariable Cox regression model of OS adjusted for age, smoking history, differentiation degree, high-risk subtype, and GGO component. Multivariable Cox regression model of DFS adjusted for age, tumor size, differentiation degree, number of N1 station examined, high-risk subtype, and GGO component.

## Discussion

Stage IA IAC is a group with high heterogeneity, nearly 20% of which suffered from recurrence after radical resection. Therefore, a nomogram of pathological stage IA IAC to predict the prognosis for individual patients is needed. In this study, we revealed the impact of GGO components and LUAD subtype on survival and developed a nomogram to predict the prognosis for individual patients based on GGO component and LUAD subtype.

In this study, the multivariate Cox analysis demonstrated that high risk subtype, which was defined as at least 5% solid or micropapillary presence, was a negative prognostic factor for patients with pathological IA IAC. In fact, many IACs have been recognized to have the mixed patterns. The same in this study, several previous studies discovered that a subtype with at least 5% solid or micropapillary presence was negatively associated with survival (Nitadori et al., 2013; Yanagawa et al., 2016; Zhao et al., 2016). Thus, we included high-risk subtype in our prognostic nomograms. In addition, we interestingly observed a different rate of gene mutation between the high-risk subtype and the low-risk subtype. After excluding the patients with unknown EGFR and ALK status, patients with low-risk subtype had an apparent high rate of EGFR gene mutation (68.3 vs. 37.0%, *p* < 0.001) and an apparent low rate of ALK rearrangement (2.2 vs. 9.6%, *p* = 0.017). Similar to our study, Villa et al. reported a high rate of EGFR mutation in the low-risk subtype in an American cohort and this finding was also discovered in a Japanese cohort (Villa et al., 2014; Yanagawa et al., 2014). Interestingly, Yoshida et al. retrospectively analyzed 61 patients treated with EGFR-Tyrosine Kinase Inhibitor (TKI) and found that the solid predominant subtype is a response predictor for EGFR-TKI (Yoshida et al., 2013). Regarding ALK rearrangement, similar results were reported in an Italian cohort (Possidente et al., 2017). Although gene status was not an independent prognostic factor and did not enter the nomogram in this study, this information is valuable when the disease recurred.

GGO components reflect a non-invasive component of tumors (Aokage et al., 2018) and several studies have proved that the presence of GGO component is associated with encouraging prognosis in early-stage patients (Hattori et al., 2017a; b; Fu et al., 2019). Consistent with numerous previous studies, we also discovered that a GGO component is a positive prognostic factor in OS and DFS for pathological stage I IAC, and a GGO component is another important variable in our prognostic nomograms. In addition, we also found that nodules with GGO component had a higher rate of EGFR gene mutation (69.8 vs. 46.7%, *p* < 0.001). Several studies have reported conflicting results. In agreement with our study, Hasegawa et al. reviewed 263 patients with LUAD in a Japanese cohort and found that patients with EGFR gene mutations had a significantly higher frequency of GGO (Hasegawa et al., 2016). A similar result was also found in a Korean cohort with 153 LUADs who had a significantly higher GGO volume percentage in EGFR exon 21 mutation subgroup (Lee et al., 2013). In contrast, Hsu et al. reported that EGFR gene mutations were detected less frequently in pure GGO nodules (Hsu et al., 2011). Regarding ALK rearrangement, there was no apparent difference in the rate of ALK rearrangement between GGO and solid nodules, and the relevant evidence is also limited. Ko et al. reported that ALK rearrangement was rare in nodules with GGO (Ko et al., 2014).

The relationship between LUAD subtype and GGO component is valuable information for treatment decisions. The details of CT characteristics can be obtained from CT image preoperatively. Understanding the relationship between LUAD subtype and GGO component can help in choosing the appropriate surgical approach. In this study, we found that patients with the low-risk subtype also had a smaller proportion of pure solid nodules and only 10% of nodules with GGO components belonged to the high-risk subtype. Moreover, 95.9% of nodules with the high-risk subtype had at least a 50% solid component. The same relationship between LUAD subtype and GGO component was also reported by Sun et al. They reviewed 1018 GGOs and discovered only 2.3% of nodules with diameter <20 mm and solid component <50% had had a micropapillary or solid component (Sun et al., 2018). These results revealed to us that 50% of CTR could be seen as a cut off when making clinical decisions. Considering that there is a significant correlation between GGO components and LUAD subtypes, we performed an interaction analysis and found no apparent interaction effect between GGO components and LUAD subtypes regarding survival. Both GGO components and high-risk subtypes had independent impacts on survival.

In the AJCC 8th TNM staging system, tumor size is the only covariate used to subdivide stage IA NSCLC. However, adopting the TNM staging system cannot accurately partition the prognosis of stage IA IAC. Previous studies have developed prognostic models including sex, age, operative approach, examined lymph nodes, vascular invasion, and EGFR gene mutation for stage IA NSCLC but ignored GGO components and LUAD subtypes (Merritt et al., 2020; Yang et al., 2020; Cai et al., 2021). To our knowledge, this study is the first to develop a prognostic nomogram based on GGO component and LUAD subtype for patients undergoing radical resection in pathological stage IA IAC, and our nomograms showed satisfactory predictive performance with an excellent Harrell’s C-index for DFS (0.667; 95% CI 0.586–0.748) and OS (0.866; 95% CI 0.841–0.891). Moreover, the variables in our nomogram are easily accessible in clinical practice.

There are some limitations that should be considered. First, this study is a retrospective study, selection bias was inevitable. Second, external validation is absent in this study. We sought help and cooperation from other institutions to verify the results. Unfortunately, there are still some difficulties, but we are actively seeking cooperation from other centers. Therefore, we only analyzed the data from our own center. We realized that the limited sample size was another limitation of our study, so we modestly referred that the practical applicability of our nomograms should be interpreted with caution. In addition, there are some patients who did not receive a gene test and no patients received NGS, which may affect the accuracy of the results about this part. The application of this result should be cautious. Finally, previous studies have reported that spread through air spaces (STAS) and maximum standardized uptake value (SUVmax) in positron emission tomography computer tomography (PET-CT) were associate with prognosis of stage IA NSCLC (Chou et al., 2021; Han et al., 2021), enrolling STAS and SUVmax should be helpful to improve this model.

In conclusion, GGO component and low risk subtype were associate with positive prognosis of patients with pathological stage IA IAC. A nomogram based on the GGO component and LUAD subtype for OS and DFS showed relatively good predictive performance. Patients with the high-risk subtype always had a nodule with at least a 50% solid component. The gene status differed according to CT characteristics of GGOs and LUAD subtype.

## Data Availability

The key raw data have been deposited into the Research Data Deposit (http://www.researchdata.org.cn), with the approval number RDDA20211153574 and the datasets used in this study are publicly available.

## References

[B1] Aokage K. Miyoshi T. Ishii G. Kusumoto M. Nomura S. Katsumata S. (2018). Influence of Ground Glass Opacity and the Corresponding Pathological Findings on Survival in Patients with Clinical Stage I Non-small Cell Lung Cancer. J. Thorac. Oncol. 13 (4), 533–542. 10.1016/j.jtho.2017.11.129 29246833

[B2] Austin J. H. Müller N. L. Friedman P. J. Hansell D. M. Naidich D. P. Remy-Jardin M. (1996). Glossary of Terms for CT of the Lungs: Recommendations of the Nomenclature Committee of the Fleischner Society. Radiology 200 (2), 327–331. 10.1148/radiology.200.2.8685321 8685321

[B3] Berry M. F. Gao R. Kunder C. A. Backhus L. Khuong A. Kadoch M. (2018). Presence of Even a Small Ground-Glass Component in Lung Adenocarcinoma Predicts Better Survival. Clin. Lung Cancer 19 (1), e47–e51. 10.1016/j.cllc.2017.06.020 28743420

[B4] Cai J.-S. Dou X.-M. Li J.-B. Yang M.-Z. Xie C.-L. Hou X. (2021). Nomogram to Predict Cancer Specific Survival in Patients with Pathological Stage IA Non-small Cell Lung Cancer. Semin. Thorac. Cardiovasc. Surg. S1043-0679, 00304-X. 10.1053/j.semtcvs.2021.06.023 34216749

[B5] Chou H.-P. Lin K.-H. Huang H.-K. Lin L.-F. Chen Y.-Y. Wu T.-H. (2021). Prognostic Value of Positron Emission Tomography in Resected Stage IA Non-small Cell Lung Cancer. Eur. Radiol. 31, 8021–8029. 10.1007/s00330-021-07801-4 33763721

[B6] Cohen J. G. Reymond E. Medici M. Lederlin M. Lantuejoul S. Laurent F. (2018). CT-texture Analysis of Subsolid Nodules for Differentiating Invasive from *In-Situ* and Minimally Invasive Lung Adenocarcinoma Subtypes. Diagn. Interv. Imaging 99 (5), 291–299. 10.1016/j.diii.2017.12.013 29477490

[B7] Ettinger D. S. Wood D. E. Aggarwal C. Aisner D. L. Akerley W. Bauman J. R. (2019). NCCN Guidelines Insights: Non-small Cell Lung Cancer, Version 1.2020. J. Natl. Compr. Canc Netw. 17 (12), 1464–1472. 10.6004/jnccn.2019.0059 31805526

[B8] Fu F. Zhang Y. Wen Z. Zheng D. Gao Z. Han H. (2019). Distinct Prognostic Factors in Patients with Stage I Non-small Cell Lung Cancer with Radiologic Part-Solid or Solid Lesions. J. Thorac. Oncol. 14 (12), 2133–2142. 10.1016/j.jtho.2019.08.002 31437531

[B9] Gao J.-W. Rizzo S. Rizzo S. Ma L.-H. Qiu X.-Y. Warth A. (2017). Pulmonary Ground-Glass Opacity: Computed Tomography Features, Histopathology and Molecular Pathology. Transl. Lung Cancer Res. 6 (1), 68–75. 10.21037/tlcr.2017.01.02 28331826 PMC5344841

[B10] Goldstraw P. Chansky K. Crowley J. Rami-Porta R. Asamura H. Eberhardt W. E. (2016). The IASLC Lung Cancer Staging Project: Proposals for Revision of the TNM Stage Groupings in the Forthcoming (Eighth) Edition of the TNM Classification for Lung Cancer. J. Thorac. Oncol. 11 (1), 39–51. 10.1016/j.jtho.2015.09.009 26762738

[B11] Han Y. B. Kim H. Mino-Kenudson M. Cho S. Kwon H. J. Lee K. R. (2021). Tumor Spread through Air Spaces (STAS): Prognostic Significance of Grading in Non-small Cell Lung Cancer. Mod. Pathol. 34 (3), 549–561. 10.1038/s41379-020-00709-2 33199839

[B12] Hasegawa M. Sakai F. Ishikawa R. Kimura F. Ishida H. Kobayashi K. (2016). CT Features of Epidermal Growth Factor Receptor-Mutated Adenocarcinoma of the Lung: Comparison with Nonmutated Adenocarcinoma. J. Thorac. Oncol. 11 (6), 819–826. 10.1016/j.jtho.2016.02.010 26917231

[B13] Hattori A. Matsunaga T. Takamochi K. Oh S. Suzuki K. (2017b). Importance of Ground Glass Opacity Component in Clinical Stage IA Radiologic Invasive Lung Cancer. Ann. Thorac. Surg. 104 (1), 313–320. 10.1016/j.athoracsur.2017.01.076 28433219

[B14] Hattori A. Matsunaga T. Takamochi K. Oh S. Suzuki K. (2017a). Prognostic Impact of a Ground Glass Opacity Component in the Clinical T Classification of Non-small Cell Lung Cancer. J. Thorac. Cardiovasc. Surg. 154 (6), 2102–2110. e2101. 10.1016/j.jtcvs.2017.08.037 28947198

[B15] Hsu K.-H. Chen K.-C. Yang T.-Y. Yeh Y.-C. Chou T.-Y. Chen H.-Y. (2011). Epidermal Growth Factor Receptor Mutation Status in Stage I Lung Adenocarcinoma with Different Image Patterns. J. Thorac. Oncol. 6 (6), 1066–1072. 10.1097/JTO.0b013e31821667b0 21512404

[B16] Ko S.-J. Lee Y. J. Park J. S. Cho Y.-J. Yoon H. I. Chung J.-H. (2014). Epidermal Growth Factor Receptor Mutations and Anaplastic Lymphoma Kinase Rearrangements in Lung Cancer with Nodular Ground-Glass Opacity. BMC Cancer 14, 312. 10.1186/1471-2407-14-312 24885886 PMC4022408

[B17] Lee H.-J. Kim Y. T. Kang C. H. Zhao B. Tan Y. Schwartz L. H. (2013). Epidermal Growth Factor Receptor Mutation in Lung Adenocarcinomas: Relationship with CT Characteristics and Histologic Subtypes. Radiology 268 (1), 254–264. 10.1148/radiol.13112553 23468578

[B18] Lortet-Tieulent J. Soerjomataram I. Ferlay J. Rutherford M. Weiderpass E. Bray F. (2014). International Trends in Lung Cancer Incidence by Histological Subtype: Adenocarcinoma Stabilizing in Men but Still Increasing in Women. Lung Cancer 84 (1), 13–22. 10.1016/j.lungcan.2014.01.009 24524818

[B19] Merritt R. E. Abdel-Rasoul M. Fitzgerald M. D’Souza D. M. Kneuertz P. J. (2021). Nomograms for Predicting Overall and Recurrence-free Survival from Pathologic Stage IA and IB Lung Cancer after Lobectomy. Clin. Lung Cancer 22, e574–e583. 10.1016/j.cllc.2020.10.009 33234491

[B20] Nitadori J.-i. Bograd A. J. Kadota K. Sima C. S. Rizk N. P. Morales E. A. (2013). Impact of Micropapillary Histologic Subtype in Selecting Limited Resection vs Lobectomy for Lung Adenocarcinoma of 2cm or Smaller. J. Natl. Cancer Inst. 105 (16), 1212–1220. 10.1093/jnci/djt166 23926067 PMC3748005

[B21] Possidente L. Landriscina M. Patitucci G. Borgia L. Lalinga V. Vita G. (2017). ALK Rearrangement in Specific Subtypes of Lung Adenocarcinoma: Immunophenotypic and Morphological Features. Med. Oncol. 34 (5), 76. 10.1007/s12032-017-0936-z 28364271

[B22] Sun F. Xi J. Zhan C. Yang X. Wang L. Shi Y. (2018). Ground Glass Opacities: Imaging, Pathology, and Gene Mutations. J. Thorac. Cardiovasc. Surg. 156 (2), 808–813. 10.1016/j.jtcvs.2018.02.110 29753514

[B23] Sung H. Ferlay J. Siegel R. L. Laversanne M. Soerjomataram I. Jemal A. (2021). Global Cancer Statistics 2020: GLOBOCAN Estimates of Incidence and Mortality Worldwide for 36 Cancers in 185 Countries. CA A. Cancer J. Clin. 71 (3), 209–249. 10.3322/caac.21660 33538338

[B24] Travis W. D. Brambilla E. Burke A. P. Marx A. Nicholson A. G. (2015). Introduction to the 2015 World Health Organization Classification of Tumors of the Lung, Pleura, Thymus, and Heart. J. Thorac. Oncol. 10 (9), 1240–1242. 10.1097/jto.0000000000000663 26291007

[B25] Travis W. D. Brambilla E. Noguchi M. Nicholson A. G. Geisinger K. R. Yatabe Y. (2011). International Association for the Study of Lung Cancer/american Thoracic Society/european Respiratory Society International Multidisciplinary Classification of Lung Adenocarcinoma. J. Thorac. Oncol. 6 (2), 244–285. 10.1097/JTO.0b013e318206a221 21252716 PMC4513953

[B26] Tsuta K. Kawago M. Inoue E. Yoshida A. Takahashi F. Sakurai H. (2013). The Utility of the Proposed IASLC/ATS/ERS Lung Adenocarcinoma Subtypes for Disease Prognosis and Correlation of Driver Gene Alterations. Lung Cancer 81 (3), 371–376. 10.1016/j.lungcan.2013.06.012 23891509

[B27] Ujiie H. Kadota K. Chaft J. E. Buitrago D. Sima C. S. Lee M.-C. (2015). Solid Predominant Histologic Subtype in Resected Stage I Lung Adenocarcinoma Is an Independent Predictor of Early, Extrathoracic, Multisite Recurrence and of Poor Postrecurrence Survival. Jco 33 (26), 2877–2884. 10.1200/jco.2015.60.9818 PMC4554749 26261257

[B28] Villa C. Cagle P. T. Johnson M. Patel J. D. Yeldandi A. V. Raj R. (2014). Correlation ofEGFRMutation Status with Predominant Histologic Subtype of Adenocarcinoma According to the New Lung Adenocarcinoma Classification of the International Association for the Study of Lung Cancer/American Thoracic Society/European Respiratory Society. Arch. Pathol. Lab. Med. 138 (10), 1353–1357. 10.5858/arpa.2013-0376-OA 24571650

[B29] Yanagawa N. Shiono S. Abiko M. Katahira M. Osakabe M. Ogata S.-y. (2016). The Clinical Impact of Solid and Micropapillary Patterns in Resected Lung Adenocarcinoma. J. Thorac. Oncol. 11 (11), 1976–1983. 10.1016/j.jtho.2016.06.014 27374456

[B30] Yanagawa N. Shiono S. Abiko M. Ogata S.-y. Sato T. Tamura G. (2014). The Correlation of the International Association for the Study of Lung Cancer (IASLC)/American Thoracic Society (ATS)/European Respiratory Society (ERS) Classification with Prognosis and EGFR Mutation in Lung Adenocarcinoma. Ann. Thorac. Surg. 98 (2), 453–458. 10.1016/j.athoracsur.2014.04.108 24961844

[B31] Yang L. Pang C. Xu F. Yang G. Xu H. Wang C. (2020). Tumor Differentiation and EGFR Mutation Associated with Disease-free Survival in Stage IA Lung Adenocarcinoma Patients with Curative Surgery. Cmar Vol. 12, 12549–12556. 10.2147/cmar.s286503 PMC7732172 33324099

[B32] Yoshida T. Ishii G. Goto K. Yoh K. Niho S. Umemura S. (2013). Solid Predominant Histology Predicts EGFR Tyrosine Kinase Inhibitor Response in Patients with EGFR Mutation-Positive Lung Adenocarcinoma. J. Cancer Res. Clin. Oncol. 139 (10), 1691–1700. 10.1007/s00432-013-1495-0 23974272 PMC11824609

[B33] Yotsukura M. Asamura H. Motoi N. Kashima J. Yoshida Y. Nakagawa K. (2021). Long-Term Prognosis of Patients with Resected Adenocarcinoma *In Situ* and Minimally Invasive Adenocarcinoma of the Lung. J. Thorac. Oncol. 16, 1312–1320. 10.1016/j.jtho.2021.04.007 33915249

[B34] Zhao Y. Wang R. Shen X. Pan Y. Cheng C. Li Y. (2016). Minor Components of Micropapillary and Solid Subtypes in Lung Adenocarcinoma Are Predictors of Lymph Node Metastasis and Poor Prognosis. Ann. Surg. Oncol. 23 (6), 2099–2105. 10.1245/s10434-015-5043-9 26842488 PMC4858562

