# Supplementary material for: Prognostic Nomograms Based on Ground Glass Opacity and Subtype of Lung Adenocarcinoma for Patients with Pathological Stage IA Lung Adenocarcinoma
Source: Front Cell Dev Biol. 2021 Dec 8;9:769881. doi: 10.3389/fcell.2021.769881 (PMC8692790; doi:10.3389/fcell.2021.769881)
Supplement: Supplementary file 1 [file Table1.DOCX]

**Supplementary table CTR of patients with high-risk subtype**

| CT Characteristics | Case NO. (%) |
| --- | --- |
| Pure GGO | 1 (1.4) |
| Part GGO CTR<50% | 2 (2.8) |
| Part GGO CTR<75% | 11 (15.3) |
| Part GGO CTR ≥75% and pure solid | 58 (80.6) |
| CT: computed tomography, GGO: ground-glass opacity, CTR: consolidation tumor ratio. | |
