# Supplementary material for: Prognostic Nomograms Based on Ground Glass Opacity and Subtype of Lung Adenocarcinoma for Patients with Pathological Stage IA Lung Adenocarcinoma
Source: Front Cell Dev Biol. 2021 Dec 8;9:769881. doi: 10.3389/fcell.2021.769881 (PMC8692790; doi:10.3389/fcell.2021.769881)
Supplement: Supplementary file 2 [file Table2.DOCX]

Point assignment and prognostic score

| Variable and prognostic score | Score | Estimated 3-year survival | Estimated 3-year survival |
| --- | --- | --- | --- |
| **OS** |  |  |  |
| Age (years) |  |  |  |
| <60 | 0 |  |  |
| 60-70 | 0 |  |  |
| >70 | 100 |  |  |
| GGO component |  |  |  |
| Positive | 0 |  |  |
| Negative | 50 |  |  |
| High-risk subtype |  |  |  |
| Positive | 6 |  |  |
| Negative | 00 |  |  |
| Total prognostic score |  |  |  |
| 40 |  | 0.99 |  |
| 110 |  | 0.95 |  |
| 140 |  | 0.90 |  |
| 180 |  | 0.80 |  |
| 200 |  | 0.70 |  |
| 70 |  |  | 0.95 |
| 100 |  |  | 0.90 |
| 160 |  |  | 0.70 |
| 190 |  |  | 0.50 |
| 210 |  |  | 0.30 |
| **DFS** |  |  |  |
| Number of N1 station examined |  |  |  |
| 0 | 100 |  |  |
| 1 | 47 |  |  |
| 2 | 38 |  |  |
| 3 | 30 |  |  |
| 4 | 20 |  |  |
| 5 | 0 |  |  |
| GGO component |  |  |  |
| Positive | 0 |  |  |
| Negative | 40 |  |  |
| High-risk subtype |  |  |  |
| Positive | 30 |  |  |
| Negative | 0 |  |  |
| Total prognostic score |  |  |  |
| 30 |  | 0.95 |  |
| 60 |  | 0.90 |  |
| 87 |  | 0.80 |  |
| 118 |  | 0.60 |  |
| 130 |  | 0.50 |  |
| 16 |  |  | 0.95 |
| 44 |  |  | 0.90 |
| 72 |  |  | 0.80 |
| 104 |  |  | 0.60 |
| 136 |  |  | 0.30 |
